# Environmentally Safe and Just Pharmacy: A Framework and Action Plan for Operating within the Earth System Boundary for Novel Entities

**DOI:** 10.1021/acs.est.5c13062

**Published:** 2026-05-07

**Authors:** Alistair B. A. Boxall, Priyanie Amerasinghe, Sally Beckenham, Michael G. Bertram, Sally Gaw, Austin D. Gray, Karen A. Kidd, Laura M. Langan, Kenneth M. Y. Leung, Jack L. Manera, Karina S. B. Miglioranza, Rik Oldenkamp, Bryan W. Brooks

**Affiliations:** † Department of Environment and Geography, 8748 University of York, York, North Yorkshire YO10 5NG, U.K.; ‡ 70306 International Water Management Institute, Battaramulla 10120, Sri Lanka; § Department of Wildlife, Fish, and Environmental Studies, 8095 Swedish University of Agricultural Sciences, Umeå 90183, Sweden; ∥ Department of Zoology, Stockholm University, Stockholm 11418, Sweden; ⊥ School of Biological Sciences, 2541 Monash University, Melbourne 3800, Australia; # School of Physical and Chemical Sciences, University of Canterbury, Christchurch 8041, New Zealand; ¶ Department of Biological Sciences, 1757 Virginia Tech, Blacksburg, Virginia 24061-0406, United States; ∇ Department of Biology, 3710 McMaster University, Hamilton, Ontario L8S 4K1, Canada; ○ Department of Environmental Health Sciences, Arnold School of Public Health, 2629 University of South Carolina, Columbia, South Carolina 29208, United States; ◆ State Key Laboratory of Marine Environmental Health and Department of Chemistry, City University of Hong Kong, Hong Kong 999077, China; & Laboratory of Ecotoxicology and Environmental Pollution, Mar del Plata University, IIMyC (CONICET), Mar del Plata 7600, Argentina; $ Amsterdam Institute for Life and Environment (A-LIFE), Faculty of Science, 1190 Vrije Universiteit Amsterdam, Amsterdam 1081, The Netherlands; @ Department of Environmental Science, 14643 Baylor University, Waco, Texas 76798, United States

**Keywords:** Active pharmaceutical ingredients, Planetary boundaries, Environmental justice, Antimicrobial resistance, Green chemistry, Ecological risk, Low- and middle-income countries

## Abstract

Active pharmaceutical ingredients (APIs) are essential for global health, yet their use and release into the environment contribute to the transgression of the Earth System Boundary for Novel Entities. This study proposes a novel framework for an Environmentally Safe and Just Pharmacy, establishing 4 overarching criteria and 12 subcriteria designed to ensure that the pharmaceutical lifecycle is environmentally safe and just from a novel entities perspective. Using the extensive expertise of our global author group, we conclude that current practices for API design and development, approval and monitoring, use and disposal are only partially or poorly aligned with our criteria. Key vulnerabilities include a lack of environmental considerations in early-stage drug design, widespread exceedances of environmental concentrations deemed safe to ecosystems, persistent selection for antimicrobial resistance in the environment, and severe data gaps in low- and middle-income countries. We further highlight environmental injustices, particularly for Indigenous and marginalized communities whose cultural identities and livelihoods are compromised by chemical contamination. To address these challenges, we present a 10-point roadmap for a transition to a sustainable future by 2050. This plan includes calls for green chemistry investments, the integration of social and cultural equity into risk assessments, and the global upgrade of treatment and management infrastructure. We emphasize that solutions to pharmaceutical impacts must be culturally sensitive and safeguard the dignity of vulnerable populations to ensure a truly just transition that operates within Earth System Boundaries.

## Graphical abstract

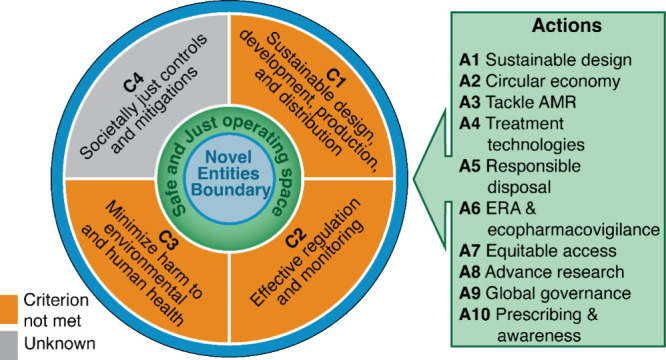

## Introduction

Pharmaceuticals are indispensable for preventing and treating diseases in both humans and animals, with over 3000 small-molecule active pharmaceutical ingredients (APIs) approved globally.^1^ These substances are released into the natural environment during manufacturing, use, and disposal. Consequently, a wide range of APIs has been detected globally in surface waters, groundwaters, and soils.^2,3^ The environmental presence of APIs has been linked to a range of impacts, including the decimation of vulture populations in South Asia,^4^ the feminization of male fish,^5^ and the selection for antimicrobial resistance (AMR) in microorganisms.^6^

In 2015, Steffen et al. proposed the Novel Entities Planetary Boundary, where Novel Entities are defined as “new substances, new forms of existing substances, and modified life forms”^7^ and which include APIs. In 2022, Persson et al. concluded that the safe operating space for novel entities is already being transgressed.^8^ The planetary boundary framework has evolved to integrate environmental justice, leading to the concept of Safe and Just Earth System Boundaries.^9–11^ Safe Earth System Boundaries define limits for maintaining a stable and resilient Earth system, while Just Earth System Boundaries ensure minimal harm to humans and serve the needs of current and future generations. Meeting both is crucial for the long-term sustainability of human society.^12^

The Earth Commission is currently in its second phase (Earth Commission 2.0, launched in 2024), shifting from conceptual development to setting actionable targets and charting pathways for society’s transition to a sustainable future. This initiative includes dedicated efforts for Novel Entities, focusing on defining their Safe and Just Earth System Boundaries and outlining pathways for a just transition. This work aims to directly contribute to these goals by proposing criteria that define Environmentally Safe and Just pharmaceuticals.

Using the extensive knowledge of the author group around the emissions, risks, and management of pharmaceuticals in the environment, we use the criteria to address the crucial question: *Is the design, manufacturing, distribution, use and disposal of pharmaceuticals currently contributing to the exceedance of the Earth System Boundary for Novel Entities?* While the focus of our analysis is on APIs and associated chemicals, such as API transformation products and production intermediates, the insights gained from transitioning pharmaceuticals toward the support of a Safe and Just Earth System Boundary for Novel Entities could also inform parallel efforts addressing other chemical classes within this special issue of *Environmental Science & Technology* and *Environmental Science & Technology Letters*. Finally, we outline a roadmap detailing actions needed to ensure the design, manufacture, use, and disposal of pharmaceuticals operate within the Novel Entities Earth System Boundary. This approach supports the Earth Commission’s vision of a just transition to a sustainable future.

## Defining Environmentally Safe and Just Pharmaceuticals

Pharmaceuticals are essential for global human, domestic animal, and livestock health, yet for these Novel Entities to be considered Environmentally Safe and Just, the production, distribution, and occurrence of these contaminants, and associated contaminants such as manufacturing intermediates, pharmaceutical metabolites, or coformulants, in the environment must not significantly harm human health or social equity, biodiversity, or ecosystem service delivery. Furthermore, any mitigating controls should not impede societal access to pharmaceuticals, particularly those on the World Health Organisation's Essential Medicines List.^13^ Here, we propose 4 overarching criteria (C) and 12 subcriteria (SC) (Table 1) that, if met, would ensure that pharmaceuticals, and associated chemicals, do not contribute to the exceedance of the Earth System Boundary for Novel Entities.

**Table 1:** A Total of 4 Criteria (C) and 12 Subcriteria (SC) That Need to Be Met to Ensure That the Pharmaceutical Lifecycle Is Environmentally Safe and Just from a Novel Entities Perspective

criterion	subcriterion
C1: Pharmaceutical design, development, production, and distribution systems are developed to reduce or eliminate harm to people and the planet.	SC1.1: Environmental safety and justice considerations are embedded into the design and development and testing processes for new pharmaceutical products.
	SC1.2: Environmental safety and justice considerations are integrated into manufacturing, formulation, and distribution systems for pharmaceuticals.
C2: Effective regulatory and postauthorization monitoring approaches are in place to protect ecological and human health.	SC2.1: Prospective ERA considers global variability in use practices, pharmaceutical accessibility, emission pathways, and ecosystem vulnerability, particularly for biodiversity hotspots and protected areas.
	SC2.2: The occurrence of the pharmaceuticals of concern in environmental compartments around the world is well understood, with data available for diverse geographies and socioeconomic settings.
	SC2.3. Ecopharmacovigilance is in place to detect, assess, understand, and prevent adverse effects of pharmaceuticals in the environment.
C3: Pharmaceuticals and associated chemicals do not cause harm to ecosystems or human health.	SC3.1: The concentrations of highly persistent active pharmaceutical ingredients and associated chemicals are kept to a minimum (i.e., used only when essential and when no safer alternatives exist).
	SC3.2: The occurrence of pharmaceuticals and associated chemicals in the environment does not cause harm to beneficial microbial, plant, and animal populations or negatively impact biodiversity or ecosystem service delivery.
	SC3.3: Pharmaceuticals and associated chemicals do not accumulate in food items to concentrations of concern to human health through direct toxicity.
	SC3.4: Pharmaceuticals and associated chemicals do not reach concentrations in drinking water of toxicological concern to human health.
	SC3.5: The presence of pharmaceuticals and associated chemicals in waste management systems and the natural environment does not result in the selection for AMR determinants.
C4: Controls and mitigations to address pharmaceutical hazards and risks are societally just.	SC4.1: Controls to minimize environmental impacts are not an obstacle for access to essential medicines and do not conflict with the Universal Declaration of Human Rights.
	SC4.2. Social, political, and cultural equity considerations are embedded into selection and implementation processes for environmental management systems for pharmaceuticals and associated chemicals.

Our criteria encompass the entire pharmaceutical lifecycle from design, development, and authorization to manufacturing, distribution, use, and ultimate disposal. They account for both direct and indirect environmental impacts, adverse effects on human health, well-being, and social equity.

The criteria recognize regional differences in regulatory frameworks, use patterns, and waste and wastewater management practices, as well as the varying vulnerabilities of people, wildlife, and ecosystem functions. We propose that the adoption of our criteria will help to ensure that environmental safety and justice considerations are better embedded into pharmaceutical design, regulation, manufacturing, use, and disposal practices so that these activities are truly safe for the planet, while simultaneously safeguarding the health and well-being of vulnerable and under-represented societies.

While we recognize that pharmaceutical lifecycles will contribute to the transgression of other Earth System Boundaries (for example, the large greenhouse gas emissions from pharmaceutical manufacturing^14^ make a significant contribution to the transgression of the Climate Change Earth System Boundary), our focus in this paper is on preventing the transgression of the Novel Entities Earth System Boundary. The paper targets pharmaceuticals and associated chemicals and does not consider other novel entity classes, such as plastics and hydrocarbons, associated with pharmaceutical packaging or distribution.

## Are We Designing, Manufacturing, Distributing, Using, and Disposing of Pharmaceuticals in an Environmentally Safe and Just Manner?

In this section, for each of the criteria and subcriteria in Table 1, we assess whether current practices for the manufacture, use, and disposal of pharmaceuticals are indeed Environmentally Safe and Just from a Novel Entities perspective. For each subcriterion, we assess whether there is either (1) alignment, (2) partial alignment, or (3) low/no alignment with the subcriterion. In instances where we believe the evidence is not yet available to come to a judgment, we conclude “unknown”. Importantly, “partial alignment” indicates the existence of some limited or regional progress only and should not be interpreted as meaning that global targets are close to being met; all criteria for which we conclude partial alignment remain significantly short of what is required from a global perspective. We additionally report the confidence we have in each classification. Where data are available across diverse geographies and socioeconomic settings, we report high confidence, whereas where major data gaps exist, particularly for low- and middle-income countries (LMICs), we report low confidence. Where the available evidence points toward alignment but with low confidence due to large knowledge gaps, we use the classification “aligned” with an explicit note of low confidence to signal that the classification may change as more data become available, especially from LMICs. Our assessments are mainly based on the extensive knowledge of the author group around pharmaceutical regulation, environmental occurrence, risks, and controls. Initial assessments of the group were also “ground-truthed” using Google Gemini to evaluate whether our assessments reflected the current knowledge and identify any major omissions. In instances where Gemini identified an omission, we obtained the suggested citations. These were read, and, where appropriate, the assessment was updated. Due to the multifaceted and complex nature of healthcare, the environment in general and pharmaceutical pollution in particular, we have not attempted to quantify the level of exceedance of each criterion, but we submit that this qualitative approach provides a robust foundation for future efforts aimed at delivering Environmentally Safe and Just Pharmacy, thus ensuring that pharmaceuticals do not contribute to the transgression of the Earth System Boundary for Novel Entities.

### C1. Pharmaceutical Design, Development, Production, and Distribution Systems Are Developed to Reduce or Eliminate Chemical Harm to People and the Planet

#### SC1.1. Environmental Safety and Justice Considerations Are Embedded into the Design, Development, and Testing Processes for New Pharmaceutical Products

The drug discovery and development process comprises six stages: target selection; hit selection and optimization; lead optimization; candidate selection; preclinical studies; clinical studies.^15^ Currently, environmental hazard and risk characteristics of APIs and coformulants are not explicitly considered in the discovery and development process, although some aspects of the process [e.g., selection of candidates that have low dose, high target specificity, aqueous solubility balanced against lipophilicity, high bioavailability, no toxicity (e.g., carcinogenic, mutagenic, reprotoxic), and no/reduced adverse effects] may result in environmental benefits.^15^ Researchers and the pharmaceutical industry are, however, actively exploring ways in which environmental hazard and risk factors can be incorporated into the pharmaceutical design process (e.g., through the work of the Innovative Health Initiative PREMIER project);^16^ however, the results of these initiatives have yet to be embedded into practice. Conclusion: Environmental hazards and risk are not currently considered in the design and development process; we conclude **low/no alignment** with high confidence.

#### SC1.2. Environmental Safety and Justice Considerations Are Integrated into Manufacturing, Formulation, and Distribution Systems for Pharmaceuticals

Manufacturing facilities, particularly those producing generic drugs, can be a key source of high concentrations of APIs and associated chemicals in the natural environment.^3,17,18^ Often, these facilities are situated in LMICs or economically disadvantaged regions which have weaker environmental oversight. This disparity fuels environmental injustice, where vulnerable populations often endure the heaviest burden of pollution and its subsequent health risks. Furthermore, these communities are frequently “location dependent”, lacking the social mobility or resources to escape contaminated areas, which traps them in a cycle of generational vulnerability.^19,20^

Advancements are being made in green chemistry and engineering to reduce emissions of harmful chemicals from manufacturing.^21^ Initiatives like the AMR Industry Alliance Manufacturing Standard are also aiming to reduce the emissions of antimicrobials to the environment.^22^ Similarly, green pharmacy practices^23,24^ have expanded over the last 2 decades. While these initiatives are being adopted, e.g., 95% of manufacturing sites owned by 21 AMRIA member companies have adopted the manufacturing standard, these and other sustainability-focused efforts are not yet universal and their application remains inconsistent across different manufacturing sites, pharmaceutical classes, producer types (e.g., innovator vs generic companies), and geographies.^25,26^ Conclusion: Green chemistry practices are being employed, and initiatives like the AMRIA standard are being adopted, but adoption of these practices is not yet universal, so we conclude **partial alignment** with high confidence. We note that this classification reflects the existence of emerging initiatives rather than widespread adoption: the majority of global manufacturing facilities, particularly generic producers in LMICs, remain outside the scope of these schemes, and the AMRIA standard covers only antimicrobials, a small fraction of pharmaceutical classes produced globally.

### C2. Effective Regulatory and Postauthorization Monitoring Approaches Are in Place to Protect Ecological and Human Health

#### SC2.1. Prospective Environmental Risk Assessment (ERA) Considers Global Variability in Use Practices, Pharmaceutical Accessibility, Emission Pathways, and Ecosystem Vulnerability, Particularly for Biodiversity Hotspots and Protected Areas

An ERA is required as part of the market authorization process for new APIs (and in select cases for APIs already on the market) in many regions of the world, including the EU, U.S., Canada, Japan, and Australia.^27^ These ERAs use exposure modeling approaches, and associated scenarios, to estimate likely concentrations in different environmental media, and these concentrations are then compared to predicted no-effect concentrations obtained using a suite of standardized ecotoxicological assays with model organisms and endpoints, using simple assessment factors that attempt to account for uncertainties (e.g., ref 28). For human-use APIs, at least, a conclusion of an unacceptable environmental risk does not currently constitute a barrier to authorization.

The conclusions from these regulatory risk assessments can likely be read across some other high-income economies (HICs) without formal risk assessments in place. They may not, however, reflect actual risks, particularly in many LMICs or disadvantaged regions in select high- and middle-income economies due to differences in pharmaceutical use patterns, release pathways, agricultural practices, and waste/wastewater collection and treatment practices, which are not reflected in the default exposure models developed for the HIC situation.^29^ Even where ERAs are available, the transparency and accessibility of ERA data can also be inconsistent.^30,31^

The lack of ERA requirements for many regions of the world is a major concern from the perspective of environmental justice because those most at risk of environmental “bads” also frequently face opacity and obstacles in trying to get information about the environmental burdens to which they are exposed. ERAs are frequently bound up with justice concerns. There is evidence to suggest that ERAs may in some contexts contribute to rather than alleviate social and environmental injustices faced by marginalized communities, particularly in the form of participatory and procedural justice.^32,33^ This is because ERA practices and protection goals may be exclusionary, for example, because of the technocratic nature of these assessments, which frames risk in very different ways to how local and especially Indigenous communities might frame harm to people and the planet, and because of the inaccessible language used in standard ERA methods (both scientific and linguistic).^32,33^ Conclusion: Prospective ERAs are required in some regions but not all, and for veterinary medicines, a negative ERA can result in restrictions on authorization. From a global perspective, we, therefore, conclude **partial alignment** with high confidence.

#### SC2.2. The Occurrence of Pharmaceuticals in Environmental Compartments around the World Is Well Understood, with Data Available for Diverse Geographies and Socioeconomic Settings

There is a growing body of research demonstrating the ubiquitous presence of APIs in surface water, groundwater, and drinking water globally. For example, the Umweltbundesamt database on pharmaceuticals in the environment includes detections in 89 countries across all UN regions,^34^ and the Global Pharmaceutical Monitoring Project detected pharmaceuticals in surface waters in 102 of the 104 countries that were monitored.^3^ In addition, the newly established Global Estuaries Monitoring (GEM) Programme under the UN Decade of Ocean Science for Sustainable Development will produce data on 64 APIs for 187 estuaries across 51 countries/regions.^35^ Over 990 APIs and associated transformation products have been detected in different environmental compartments and matrices.

Given that there are 195 recognized countries in the world and more than 3000 small-molecule APIs that are approved,^1^ our understanding is still far from “well understood”, with no data existing for a number of countries and for many pharmaceuticals that are in widespread use. Furthermore, only limited data are available for some compartments (e.g., air, soil, and food items are less studied). Data are mainly available from more developed regions, particularly from China, Europe, and the United States, with considerable gaps in most LMICs, making it currently not possible to draw a truly global, comprehensive picture of occurrence, especially factoring in diverse socioeconomic settings.

From a justice viewpoint, those most likely to be affected by contaminated surface water, groundwater, and drinking water are in poor communities in the global south who are already facing the impacts of marginalization and compounded vulnerabilities. For example, the gendered burden of water insecurity is such that, in Sub-Saharan Africa, 766 million people (approximately 60% of the population) lack clean uncontaminated drinking water,^36^ and this disproportionately affects women and girls. In 53 countries where the data exist, women and girls spend 250 million hours per day on water collection, over 3 times more than men and boys.^37^ This disproportionately exposes them to many environmental determinants of disease. Conclusion: Data are not available for all countries, and the data that are available are predominantly for aquatic systems, so we conclude **partial alignment** with high confidence.

#### SC2.3. Ecopharmacovigilance Is in Place to Detect, Assess, Understand, and Prevent Adverse Effects of Pharmaceuticals in the Environment

The catastrophic decline in vulture populations in regions of South Asia in the late 1990s due to the use of diclofenac, a nonsteroidal anti-inflammatory pharmaceutical,^4^ serves as a warning that unexpected impacts can and do occur from the use of pharmaceuticals. While some regions, such as Europe, and some U.S. states have environmental monitoring and reporting approaches in place that could pick up these unexpected impacts (e.g., ecological monitoring under the EU Water Framework Directive or the Wildlife Incident Surveillance Scheme in the U.K.), proactive monitoring systems aimed at pharmaceuticals are not yet in place around the world. The concept of ecopharmacovigilance (EPV), where monitoring approaches for API safety are extended to detect impacts on the natural environment, is, however, gaining traction, with some from the pharmaceutical industry (e.g., AstraZeneca^38^) implementing programs to review emerging environmental data on their products (e.g., 27). However, EPV is not universally mandated nor consistently implemented globally. It is often “targeted” at the highly researched substances rather than considering pharmaceuticals in general.^39^ Systematic postmarket environmental data collection and public sharing are still limited. Where EPV schemes identify unforeseen adverse environmental effects, these findings should feed back into the licensing approval process, analogous to how postmarket human safety signals trigger regulatory re-evaluation of approved medicines. Conclusion: Ecological monitoring schemes do not yet specifically consider pharmaceuticals, and company-level ecopharmacovigilance schemes are rare, so we conclude **low/no alignment** with high confidence.

### C3. Pharmaceuticals and Associated Chemicals Do Not Cause Harm to Ecosystems or Human Health.

#### SC3.1. The Concentrations of Highly Persistent Active Pharmaceutical Ingredients and Associated Chemicals Are Kept to a Minimum (i.e., Used Only When Essential and When No Safer Alternatives Exist)

Cousins et al.^40^ argue that the release of highly persistent chemicals will result in increasing probabilities of the occurrence of known and unknown effects of a substance. They warn that, once adverse effects are identified, it could take decades or longer to reverse the contamination. Restriction or minimization (e.g., using the essential use approach) of the emissions of highly persistent active ingredients and associated chemicals is therefore not only precautionary but could serve to prevent long-term impacts of pharmaceuticals, and associated chemicals, that are hard to reverse. Most pharmaceuticals are designed for resistance to degradation because this ensures the delivery of therapeutic benefits, predictable elimination kinetics, and a reduced likelihood of side effects. This inherent stability means they often do not fully degrade in conventional wastewater treatment plants (WWTPs) or in the natural environment, leading to their ubiquitous presence in surface waters, soils, and sediments.^3,34^ When contemporary regulatory cutoff values are considered, many pharmaceuticals are classified as persistent, having long degradation half-lives in water;^41^ in addition, a few active ingredients, including widely used substances like fluoxetine and atorvastatin, have even been defined as per- and polyfluoroalkyl substances (PFAS), or “forever” chemicals.^42^ Conclusion: Controls do not yet exist for highly persistent pharmaceuticals, so we conclude **low/no alignment** with high confidence.

#### SC3.2. The Occurrence of Pharmaceuticals and Associated Chemicals in the Environment Does Not Cause Irreversible Harm to Beneficial Microbial, Plant, and Animal Populations or Negatively Impact Biodiversity or Ecosystem Service Delivery

The occurrence of pharmaceuticals in the environment has been reported to result in a wide range of effects at different organismal levels.^43^ Examples include the impacts of diclofenac on vulture populations in South Asia,^4^ the collapse of a fish population^5^ and subsequent indirect effects on an aquatic food web^44^ due to exposure to an estrogen agonist, and behavioral changes in fish and other aquatic wildlife.^45–47^ Concerns have also been raised over potential impacts on the delivery of key ecosystem services including disease regulation,^48^ pollutant storage and filtration,^49,50^ and nitrogen and carbon cycling.^51,52^

Comparison of the concentration data from a global river-monitoring study with predicted no-effect concentrations derived from standard ecotoxicological endpoints for fish, invertebrates, and algae, which are routinely used in traditional ERAs, and nonstandard endpoints that are linked to adverse outcomes suggest that around 44% of locations globally have concentrations of pharmaceuticals of concern for ecological health.^53^ While the locations of greatest concern are concentrated in LMICs, even better-regulated regions have areas identified as at risk from API exposure. This is viewed as a growing and major threat to biodiversity and ecosystems.^54,55^ Chronic exposure to mixtures of pharmaceuticals,^56^ even at low concentrations, can have complex and potentially irreversible impacts on reproductive health, development, ecosystem functions, and biodiversity, which are still not fully understood but represent a significant concern.^57–59^ Notably, SC3.2 addresses the retrospective dimension of ERA, that is, whether pharmaceuticals currently in use and present in the environment cause harm, complementing the prospective ERA requirements in SC2.1. Conclusion: Given the large number of locations globally that have concentrations of pharmaceuticals of ecological concern, we conclude **low/no alignment** with high confidence.

#### SC3.3. Pharmaceuticals and Associated Chemicals Do Not Accumulate in Food Items to Concentrations of Concern to Human Health through Direct Toxicity

APIs can be taken up by crops (from contaminated soil or irrigation with reclaimed wastewater)^60^ and can be present in animal products (from veterinary use or environmental exposure).^61^ Moreover, biomonitoring studies have demonstrated the transfer of APIs, such as the anticonvulsant carbamazepine, from food into humans.^62^ As part of food safety surveillance, pharmaceuticals commonly used in farm animals, such as antibiotics, are usually routinely monitored to safeguard human health.^63^ Studies generally suggest that the levels detected in food items are low and unlikely to pose an immediate acute risk to human health.^64^ However, some individuals can be hypersensitive to some classes of pharmaceuticals,^65^ and adverse effects from chronic, low-level dietary exposure to a mixture of pharmaceutical residues may also be possible. The risks from long-term, low-level exposures are also yet to be fully understood. Consumption of food sourced from the wild, which is particularly relevant to LMICs, could also result in high levels of exposure. Conclusion: The available evidence, primarily from well-monitored settings, suggests that risks to human health from food exposure are low; we therefore conclude **alignment** but with low confidence. Confidence is low due to very large data and knowledge gaps, particularly regarding wild food consumption, chronic mixture exposure, and food systems in LMICs; the classification may change substantially as more data become available.

#### SC3.4. Pharmaceuticals and Associated Chemicals Do Not Reach Concentrations in Drinking Water of Toxicological Concern to Human Health

Trace levels of pharmaceuticals are frequently detected in drinking water worldwide.^66^ Regulatory bodies such as the World Health Organization generally state that these detected levels are typically very low (often nanograms per liter) and unlikely to pose a significant acute health risk to the general human population.^67^ However, concerns still remain, particularly for vulnerable populations (e.g., infants, pregnant women, and elderly), and around the potential long-term, cumulative effects of exposure to mixtures of various compounds over a lifetime.^67^ Studies into the fate of iodinated contrast media during drinking water treatment show that these substances can be transformed by chlorination, producing cytotoxic and genotoxic transformation products,^68^ highlighting the potential for API transformation reactions to increase risks to human health. Monitoring of finished drinking waters and source waters is inconsistent globally, particularly for groundwater resources impacted by urban waste streams, and some less-well-studied regions may have higher concentrations due to poor source-water quality and/or inadequate treatment. Conclusion: The available evidence, primarily from well-monitored settings, suggests that risks to human health from drinking water exposure are low; we therefore conclude **alignment** with low confidence. Confidence is low because drinking water monitoring focuses on parent compounds and not transformation products and is highly inconsistent globally; in many LMICs where groundwater is a primary source and treatment infrastructure is limited, the evidence base is insufficient to sustain this classification, and risks may be substantially higher than current data suggest.

#### SC3.5. The Presence of Pharmaceuticals and Associated Chemicals in Waste Management Systems and the Natural Environment Does Not Result in the Selection of AMR Determinants

There is strong and growing scientific consensus that antimicrobials and other pharmaceuticals select for AMR in microorganisms and bacteriophages within waste (water) systems and the environment.^6,69,70^ These contaminants exert this selection pressure in concerted action with their biologically active metabolites and transformation products, along with other medications^70^ and nonpharmaceutical chemicals such as metals.^71^ This exacerbates the role of the natural environment as a critical pathway for exposure to antibiotic- and antifungal-resistant microorganisms,^72^ AMR genes, and subsequently associated mobile genetic elements,^73^ contributing to the global AMR crisis. Originating from manufacturing discharge and human and animal excretion, antimicrobials and AMR determinants enter wastewaters, surface waters, biosolids^74^ and soils^75^ and, ultimately, find their way back to humans.^76^

Various experimental and clinical data-driven approaches have been applied to derive predicted no-effect concentrations for resistance (PNEC_R_) for antibiotics. PNEC_R_s aim to represent “safe” concentrations below which antibiotics do not contribute to AMR selection. PNEC_R_s vary widely depending on the method applied, but those based on clinical minimum inhibitory concentrations (MICs) appear to be more conservative than experimentally derived PNEC_R_s.^77^ Comparison of antibiotic occurrence data from the global monitoring efforts with MIC-based PNEC_R_s proposed by the AMR Industry Alliance indicates that 17% of the surface waters sampled across all continents have concentrations of at least one antibiotic of concern for AMR selection,^78^ and such exceedances are predicted to increase with the growing extraction of water and with climate change.^79^ Conclusion: Given the widespread occurrence of antibiotics at concentrations above those believed to be safe for resistance selection, we conclude **low/no alignment** with high confidence.

### C4. Controls and Mitigations to Address Hazards and Risks of Pharmaceuticals and Associated Chemicals Are Societally Just

#### SC4.1. Controls to Minimize Environmental Impacts Are Not an Obstacle to Access to Essential Medicines and Do Not Conflict with the Universal Declaration of Human Rights

Around 2 billion people currently still do not have access to essential medicines, in particular vulnerable populations in LMICs.^80^ Although incentives for innovation can catalyze diverse benefits for public health and the environment, stricter environmental controls could increase production costs, which might impact the affordability and availability of essential medicines, especially in LMICs. Stricter controls could result in a move toward sourcing pharmaceuticals from cheaper, unregulated, and less safe supply chains. Similarly, patent restrictions could also determine the costs and limit the availability of more environmentally benign pharmaceuticals in LMICs.^81^ While the United Nations Sustainable Development Goals aim to achieve both environmental sustainability and equitable access to healthcare, realizing this balance while avoiding societal, economic, and environmental risk trade-offs represents an ongoing challenge that requires careful policy design, international cooperation, and potentially new funding mechanisms for the development and distribution of essential medicines to ensure that environmental measures do not inadvertently hinder access to life-saving pharmaceuticals. Furthermore, many LMICs currently lack adequate infrastructure for managing pharmaceutical waste, meaning that without targeted investment and support, the absence of environmental controls may secure short-term access to medicines while creating long-term environmental harm, a trade-off that demands careful attention in any just and sustainable framework. Conclusion: While a significant number of people do not have access to essential medicines, the influence of environmental controls is not understood, so we conclude **unknown** with low confidence.

#### SC4.2. Social, Political, and Cultural Equity Considerations Are Embedded into the Selection and Implementation Processes for Environmental Management Systems for Pharmaceuticals and Associated Chemicals

The shift toward environmentally safe pharmaceuticals must integrate a robust environmental justice framework that accounts for the diverse cultural, spiritual, political, and socio-economic realities of global communities. For many Indigenous and other local populations, the environment is not merely a resource but a foundational source of identity; pharmaceutical residues in these ecosystems represent a potential violation of relational values, often exacerbated by a lack of political agency.^82^

Consequently, management approaches must be culturally sensitive and socially inclusive, based on principles of participatory development.^83^ A transition toward stricter standards must be balanced with the need to safeguard the livelihood dependencies of vulnerable populations.^84^ Abrupt regulatory shifts or the closure of noncompliant facilities risk displacing workers, particularly in the generic manufacturing sector, who rely on these roles for survival. Without social protections and alternative economic pathways, solutions to the impacts of APIs may inadvertently deepen the poverty of the most left behind. To realize the UN Sustainable Development Goals, pharmaceutical management must protect the biosphere while upholding the dignity and prosperity of those historically excluded from industrial progress. Conclusion: While environmental management approaches are in place in some regions and sectors for pharmaceuticals, the wider societal impacts of these approaches are not clear so we conclude **unknown** with low confidence.

Overall, we conclude that current practices for the design, distribution, use, and disposal of pharmaceuticals do not currently meet most of the criteria for being Environmentally Safe and Just from a Novel Entities perspective (Figure 1). The global use of pharmaceuticals is, therefore, contributing to the transgression of the Earth System Boundary for Novel Entities. Our approach has been qualitative, so studies quantifying by how much our criteria are being exceeded will be very useful for risk management purposes. While awareness is increasing and progress is being made in areas like green and sustainable chemistry and engineering and ERA practice, significant challenges persist. There is substantial evidence of harmful environmental and social impacts from manufacturing, widespread and persistent environmental contamination, and a clear contribution to the AMR crisis. Addressing these issues, particularly in the context of environmental justice and ensuring equitable access to pharmaceuticals, requires concerted, globally coordinated efforts, and significant investment. A critical equity dimension is the increasing outsourcing of pharmaceutical manufacturing to LMICs, which bear a disproportionate pollution burden from supplying high-income countries that consume the bulk of global pharmaceutical production yet often take little action to address their LMIC environmental footprint. This geopolitical dimension of pharmaceutical supply chains must be considered in the development and implementation of any just framework, ensuring that efforts to address environmental impacts in one part of the world do not displace or exacerbate burdens elsewhere. Many aspects are partially aligned with our criteria or still fall into the “unknown” category due to knowledge gaps, especially concerning regulation, human health effects, and justice. For SC3.3 and SC3.4, the available evidence suggests alignment with the criteria for human health risks from food and drinking water but with low confidence given the limited geographic coverage of research into human health risks of pharmaceuticals, particularly in LMICs. These classifications may, therefore, not hold globally as more data become available. In the next section, we describe a roadmap for bringing pharmaceuticals to safe and just operating space.

**Figure 1:**
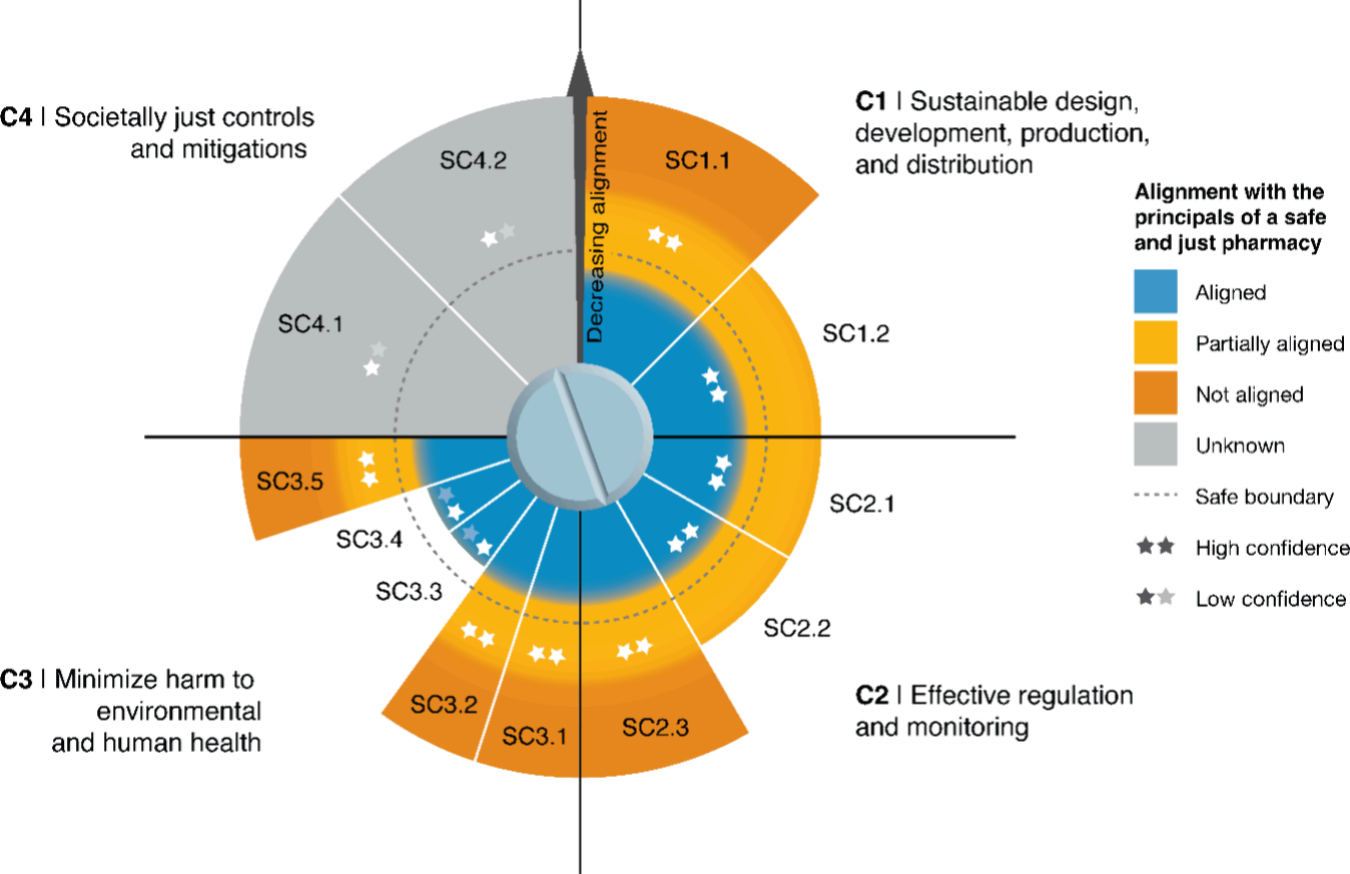
Current status of the 4 overarching criteria (C1–C4) and 12 subcriteria for Environmentally Safe and Just pharmaceuticals. Each criterion occupies one quartile of the diagram, and each slice represents one subcriterion. Slice length indicates the degree of alignment: slices contained within the dashed black safe boundary are aligned; slices extending just beyond it are partially aligned; slices at full extent are not aligned or are unknown. Color indicates classification: blue = aligned; yellow = partially aligned; orange = not aligned; gray = unknown. Stars within each slice indicate confidence in the classification (★★ = high confidence, reflecting a strong and consistent evidence base across diverse settings; ★☆ = low confidence, reflecting major data gaps, particularly for LMICs). The safe boundary represents the threshold below which pharmaceuticals would not contribute to the transgression of the Novel Entities Earth System Boundary. Note that “partially aligned” indicates limited or regional progress only and should not be interpreted as meaning global targets are close to being met and that “aligned” classifications with low confidence (★☆) are based primarily on evidence from well-monitored settings and may not apply globally.

## Toward 2050: A Suggested Roadmap for Bringing Pharmaceuticals into a Safe and Just Operating Space

Addressing the environmental impacts of APIs and associated chemicals in a just manner will require a multifaceted approach involving local through to global cooperation among governments, the pharmaceutical industry, water and waste utilities, healthcare providers, nongovernmental organizations, the research community, and the public. Below, we outline 10 key actions that we recommend should be advanced as a global society to realize an Environmentally Safe and Just Pharmacy in the future. The primary stakeholder groups that will need to be engaged in each set of actions are identified. The interlinkages of each of these actions to the subcriteria are shown in Figure 2.

**Figure 2:**
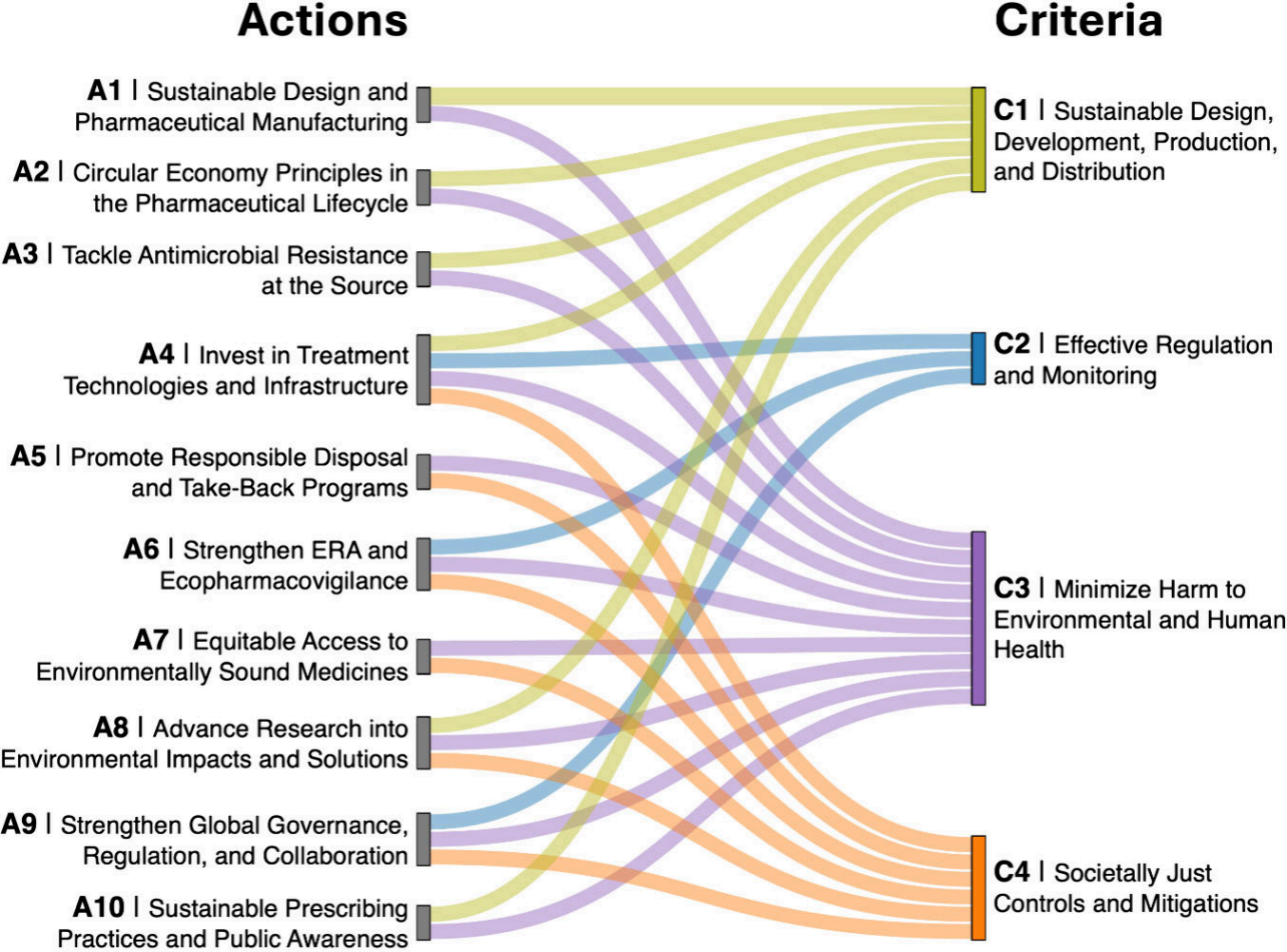
Suggested roadmap for achieving an Environmentally Safe and Just Pharmacy by 2050. A total of 10 priority actions (A1–A10) are presented and linked to the 4 overarching criteria (C1–C4) for Safe and Just pharmaceuticals. The linkage diagram illustrates how each action contributes to addressing specific criteria, highlighting the pathways toward reducing environmental impacts while ensuring equitable access to medicines.

### Upstream Prevention and Design (Pharmaceutical Industry)

#### A1. Strategically Invest in Sustainable Molecular Design and Implement Green Chemistry and Engineering Principles in Pharmaceutical Manufacturing

By design, pharmaceutical products that are developed with lower environmental impacts will significantly reduce unjust burdens downstream.^85^ This consideration will also address the challenge of pollution from pharmaceutical manufacturing and assist with transitions from existing medications to more sustainable therapeutics. We should globally promote the development of “benign by design” pharmaceuticals that are inherently less harmful to the environment and promote the use of greener synthesis routes, minimizing hazardous reagents, reducing waste generation, decreasing accidents, and improving energy and water efficiency throughout the manufacturing process.

#### A2. Foster Circular Economy Principles in the Pharmaceutical Lifecycle

Embracing systems thinking and moving away from a linear “take-make-waste” model would reduce waste, conserve resources, and lower the overall environmental footprint of the healthcare industry. We should continue to learn from Indigenous Knowledge systems, such as whakapapa, a Ma̅ori cultural framework for a “way of knowing”, to map the full lifecycle of pharmaceuticals, including the origins of natural resources extracted to develop chemical feedstocks used in manufacturing and distribution.^86^ We should also innovate and implement circular economy models for pharmaceutical solvents and other chemicals used in manufacturing. This action includes increasing recycling, promoting reusable packaging where safe and feasible, and finding innovative ways to repurpose byproducts.

#### A3. Tackle AMR at the Source

Environmental release of antibiotics is a major driver of AMR, which is one of the leading global health crises of our time. Reducing the environmental inputs of these Novel Entities is paramount for human health, biodiversity, and ecosystem services. We should implement stringent controls on antibiotic emissions from all sources, including manufacturing effluent, hospital discharges, and WWTP effluents, globally to prevent the release of APIs and associated chemicals that contribute to AMR. We should also promote responsible antibiotic use for human and animal health to reduce overall consumption and subsequent environmental release.^63^ Broader adoption of new tools and stewardship approaches like those developed by the AMR Industry Alliance (https://www.amrindustryalliance.org/) including implementation of targeted water quality guidelines and development of nontraditional partnerships with government agencies, academics, and nongovernmental organizations, needs to be prioritized for planetary health.

### Management, Treatment, and Regulation (Healthcare Providers, Water Utilities, Policy Makers, and Regulators)

#### A4. Invest in Treatment Technologies and Infrastructure

A large portion of APIs and associated chemicals enter the environment via wastewater. Improved pharmaceutical treatability and treatment technologies are essential to reduce environmental exposure and protect aquatic ecosystems and associated beneficial uses, including potable water supplies. In countries with high wastewater connectivity, we should upgrade WWTPs with technologies specifically designed to remove pharmaceutical residues (e.g., advanced oxidation processes, activated carbon filtration, membrane, and surface materials targeting chemical classes). In LMICs and other disadvantaged areas, where current treatment capabilities are often insufficient and where wastewater connectivity is low, we should explore the use of low-cost, low-maintenance, and decentralized solutions^87^ that reduce the environmental burden from pharmaceuticals. This would also help to address a range of other social-justice issues. While these social benefits derive primarily from improved sanitation rather than directly from the removal of pharmaceutical residues, they represent important cobenefits of investment in decentralized water and sanitation infrastructure. This includes, for example, gender inequality (evidence shows that women and girls in many global south contexts face harassment and sexual violence as a direct result of poor sanitation systems^88^), school absenteeism (from children suffering from sanitation-related diseases), and economic mobility (because time poverty for the poor, especially women, is partly caused by sickness from poor sanitation^89^). For example, nature-based solutions, such as constructed wetlands and bank infiltration, including horizontal levee systems, should be expanded to all countries with full and informed participation from local and national governments, local communities, and other stakeholders and prioritized on the basis of indices, such as the Multidimensional Poverty Index,^90^ that highlight the areas most in need of intervention, regardless of country developmental status. Similarly, ongoing advances in transitioning to more decentralized^91^ and distributed wastewater technologies promise transformational benefits for more sustainable water reuse practices in some situations.

#### A5. Promote Responsible Disposal and Take-Back Programs for Unused/Expired Pharmaceuticals

Incorrect disposal of unused medications directly contributes to pharmaceutical pollution and presents risks for public health. We should establish and widely publicise accessible and convenient take-back programs (e.g., https://www.dea.gov/takebackday) for unused and expired pharmaceuticals globally, expand partnerships with pharmacies (e.g., ref 92), and educate healthcare professionals and the public on proper disposal methods, such as hazardous waste incineration or the use of novel in situ treatment methodologies such as pyrolysis,^93^ to prevent flushing or landfilling, especially in areas lacking appropriate waste management.

#### A6. Strengthen Global ERA and Ecopharmacovigilance

A more geographically inclusive approach to ERA, ecopharmacovigilance, and environmental monitoring will help to minimize the use of environmentally harmful pharmaceuticals and associated chemicals and, once a new pharmaceutical is approved, identify associated environmental problems at an early stage. We should develop globally inclusive and more comprehensive ERAs that consider real-world use practices, diverse environmental conditions (especially in vulnerable ecosystems), and potential additive, antagonistic, and synergistic effects of pharmaceutical mixtures. We should establish and implement robust ecopharmacovigilance and environmental monitoring programs within which spatially explicit chemical mapping is realized, where global production, distribution, consumption, and disposal practices are documented postmarket, and transparent data sharing facilitates monitoring of global occurrence and of adverse impacts for/from pharmaceuticals in the environment.

### Equity, Governance, and Research (Policy Makers and Research Community)

#### A7. Ensure Equitable Access to Environmentally Sound Pharmaceuticals and Practices

Environmental justice is crucial for all people in all countries; it essentially represents the “North Star” for public health and environmental stewardship. Sustainability efforts must not compromise the fundamental human right to health and access to medicines nor should they exacerbate existing inequalities. We should, therefore, develop policies that balance achieving the human right to a healthy environment, including equitable access to essential medicines, particularly for vulnerable populations and LMICs. This action includes exploring innovative financing models, technology transfer, and open-source approaches for greener pharmaceutical production. We must ensure that environmental controls do not disproportionately burden or negatively impact vulnerable societies.

#### A8. Advance Research into Environmental Impacts and Solutions

Considerable knowledge gaps still remain around pharmaceuticals in the environment.^94^ Continued research is vital to fully understanding the problem and developing effective and innovative solutions. Increased funding is needed for independent research on the long-term, low-dose, and mixture effects of pharmaceuticals on biodiversity, ecosystem services, and human health. Investments are needed to design less hazardous chemicals and materials and to develop novel waste and wastewater treatment techniques and ecofriendly alternatives. Given the importance of the social, cultural, and economic factors influencing pharmaceutical supply, use, and disposal, research into these areas is required. To maximize the impact for the future, we need to establish forward-looking global coalitions that include strategic investment models with contributions from diverse stakeholders, particularly given the regional unpredictability of government funding mechanisms and shifting priorities associated with political pendulum swings. We need to be more agile so that key developments and findings from the research community can be more rapidly embedded into policy and practice.

#### A9. Strengthen Global Governance, Regulation, and Collaboration

The environmental impact of pharmaceuticals is inherently a transboundary issue, in which they are often produced with chemical feedstocks from different regions and then consumed by human populations in other global regions. In 2015, environmentally persistent pharmaceutical pollutants were adopted as an emerging policy issue at the Fourth International Conference of Chemicals Management as part of the Strategic Approach to International Chemicals Management (SAICM) policy framework. In 2023, the Global Framework on Chemicals—for a Planet Free of Harm from Chemicals and Waste was adopted as the successor to SAICM. In June 2025, a new Intergovernmental Science-Policy Panel on Chemicals, Waste and Pollution was established under the mandate of the United Nations Environment Assembly (https://www.unep.org/isp-cwp). Looking forward, we should develop and harmonize international frameworks on pharmaceutical environmental performance, from development and manufacturing standards to postconsumer waste management, and we need to foster greater collaboration on this issue among researchers, governments, regulatory bodies, industry, nongovernmental and international organizations (e.g., UNEP, WHO, UNDRR, and UNDP), and civil society.

#### A10. Promote Sustainable Procuring and Prescribing Practices and Raise Public Awareness

Patient use and prescribing habits contribute significantly to the environmental concentrations of pharmaceuticals. The public and prescriber behaviors that lead to pharmaceutical use and misuse are a result of modern healthcare systems being inherently and intricately reliant on pharmaceuticals. Systemic reform is needed to tackle the challenge of pharmaceuticals in the environment. Advances in precision medicine promise opportunities to more intentionally prevent, diagnose, and treat illnesses and diseases in transformational ways that decrease per capita pharmaceutical consumption. We should also educate healthcare providers on the environmental impact of their prescribing choices, encouraging “eco-directed sustainable prescribing” where appropriate (e.g., considering environmental profiles of pharmaceuticals and avoiding overprescription^95^). Further, we should develop nontraditional partnerships with related disciplines (e.g., community health education specialists) to raise public awareness about the environmental implications of pharmaceutical use and the importance of responsible consumption and disposal. We should promote public health campaigns for health-enhancing behaviors and health-promoting environments to reduce the burden of noncommunicable diseases and the associated pharmaceutical burden.

## Looking Forward

To prevent the contribution of the pharmaceutical lifecycle to the transgression of the Novel Entities Earth System Boundary, we must work together so that global society can benefit from equitable access to pharmaceuticals while protecting planetary health. We believe that our criterion-based framework, assessment, and proposed actions provide a strong foundation from which to deliver change towards more sustainable healthcare. Looking forward, the broader community, including policy makers, regulatory authorities, the pharmaceutical industry, the waste and wastewater management sector, non-governmental organisations, and researchers and research funders (including philanthropic organisations), needs to work together to explore how our proposed actions can be implemented. This work will need to identify the existing barriers (e.g., regulatory and economic) and challenges associated with the implementation of each action and the potential disbenefits. The work will need to explore different options (e.g., changes in policy or regulation or voluntary initiatives) for the delivery of each action and the relevant scales (local, regional, national, and global) for each action. Finally, it will likely be impossible to deliver all actions at once, so our actions need to be systematically prioritized with initial efforts focused on areas and activities that are going to result in the biggest wins for the planet and society. We anticipate that lessons learned from initiatives aimed at designing and developing environmentally safe and just pharmaceuticals for the future will continue to result in innovation cobenefits for other classes of Novel Entities. Global adoption of the 10 actions presented here will support the Earth Commission’s ambition of ensuring that the Novel Entities Earth System Boundary is not transgressed and a Safe and Just transition towards a sustainable pharmacy future is realized for all people. Given the complex and evolving nature of the Novel Entities planetary boundary and the substantial uncertainties that remain in our understanding of pharmaceutical risks and impacts, all assessments and recommendations presented here should be continuously updated and re-evaluated as new scientific information, data, and concepts emerge. We also recognize that this set of actions represents a starting point rather than a definitive roadmap; the implementation of our recommended actions will require the involvement of a much wider range of stakeholders, including from industry, government, civil society, and affected communities, and each action will itself require extensive review and evaluation, particularly with regards to justice implications, before being implemented. We strongly believe, however, that this framework serves as an important foundation from which to open discussions and begin to make tangible progress on this pressing planetary health challenge.
